# Unifying Theories of Psychedelic Drug Effects

**DOI:** 10.3389/fphar.2018.00172

**Published:** 2018-03-02

**Authors:** Link R. Swanson

**Affiliations:** ^1^Center for Cognitive Sciences, University of Minnesota, Minneapolis, MN, United States; ^2^Department of Philosophy, University of Minnesota, Minneapolis, MN, United States; ^3^Minnesota Center for Philosophy of Science, University of Minnesota, Minneapolis, MN, United States

**Keywords:** psychedelic drugs, LSD, psilocybin, ego dissolution, cognitive flexibility, entropic brain theory, integrated information theory, predictive processing

## Abstract

How do psychedelic drugs produce their characteristic range of acute effects in perception, emotion, cognition, and sense of self? How do these effects relate to the clinical efficacy of psychedelic-assisted therapies? Efforts to understand psychedelic phenomena date back more than a century in Western science. In this article I review theories of psychedelic drug effects and highlight key concepts which have endured over the last 125 years of psychedelic science. First, I describe the subjective phenomenology of acute psychedelic effects using the best available data. Next, I review late 19th-century and early 20th-century theories—*model psychoses theory, filtration theory*, and *psychoanalytic theory*—and highlight their shared features. I then briefly review recent findings on the neuropharmacology and neurophysiology of psychedelic drugs in humans. Finally, I describe recent theories of psychedelic drug effects which leverage 21st-century cognitive neuroscience frameworks—*entropic brain theory, integrated information theory*, and *predictive processing*—and point out key shared features that link back to earlier theories. I identify an abstract principle which cuts across many theories past and present: psychedelic drugs perturb universal brain processes that normally serve to constrain neural systems central to perception, emotion, cognition, and sense of self. I conclude that making an explicit effort to investigate the principles and mechanisms of psychedelic drug effects is a uniquely powerful way to iteratively develop and test unifying theories of brain function.

## Introduction

Lysergic acid diethylamide (LSD), N,N-dimethyltryptamine (DMT), psilocybin, and mescaline—the ‘classic’ psychedelic drugs—can produce a broad range of effects in perception, emotion, cognition, and sense of self. How do they do this? Western science began its ‘first wave’ of systematic investigations into the unique effects of mescaline 125 years ago. By the 1950s, rising interest in mescaline research was expanded to include drugs like DMT, LSD, and psilocybin in a ‘second wave’ of psychedelic science. Because of their dramatic effect on the character and contents of subjective awareness, psychedelic drugs magnified the gaps in our scientific understanding of how brain chemistry relates to subjective experience (see Evarts, 1957; Purpura, 1968). Huxley (1991, p. 12) commented that our understanding circa 1954 was “absurdly inadequate” and amounted to a mere “clue” that he hoped would soon develop into a more robust understanding. “Meanwhile the clue is being systematically followed, the sleuths—biochemists, psychiatrists, psychologists—are on the trail” (Huxley, 1991, p. 12). A ‘third wave’ of psychedelic science has recently emerged with its own set of sleuths on the trail, sleuths who now wield an arsenal of 21st-century scientific methodologies and are uncovering new sets of clues.

Existing theoretical hurdles span five major gaps in understanding. The first gap is that we do not have an account of how psychedelic drugs can produce such a broad diversity of subjective effects. LSD, for example, can produce subtle intensifications in perception—or it can completely dissolve all sense of space, time, and self. What accounts for this atypical diversity?

The second gap is that we do not understand how pharmacological interactions at neuronal receptors and resulting physiological changes in the neuron lead to large-scale changes in the activity of neural populations, or changes in brain network connectivity, or at the systems-level of global brain dynamics. What are the causal links in the multi-level pharmaco-neurophysiological chain?

The third gap is that we do not know how psychedelic drug-induced changes in brain activity—at any level of description—map onto the acute subjective phenomenological changes in perception, emotion, cognition, and sense of self. This kind of question is not unique to psychedelic drugs (i.e., Crick and Koch, 1998; Tononi and Edelman, 1998) but our current understanding of psychedelic drug effects clearly magnifies the disconnect between brain science and subjective experience.

Fourth, there is a gap in our understanding of the relationships between psychedelic effects and symptoms of psychoses, such as perceptual distortion, hallucination, or altered self-reference. What is the relationship between psychedelic effects and symptoms of chronic psychotic disorders?

Fifth and finally, there is a gap in our clinical understanding of the process by which psychedelic-assisted therapies improve mental health (Carhart-Harris and Goodwin, 2017). Which psychedelic drug effects (in the brain or in subjective experience) enable clinical improvement? How?

Scientific efforts to understand diverse natural phenomena aim to produce a single theory that can account for many phenomena using a minimal set of principles. Such theories are sometimes called *unifying theories*. Not everyone agrees on the meaning of ‘unification’ or ‘unifying theory’ in science.^1^ Morrison (2000) observed that, although theory unification is a messy process which may not have discernible universal characteristics, historically successful unifying scientific theories tend to have two common features: (1) a *formalized framework* (quantitative mathematical descriptions of the phenomena) and (2) *unifying principles* (abstract concepts that unite diverse phenomena). On this conception, then, a unifying theory of psychedelic drug effects would offer a single formalized (mathematical or computational) framework capable of describing diverse psychedelic phenomena using a minimal set of unifying principles. Unfortunately, the survey of literature in this review does not locate an existing unifying theory of psychedelic drug effects. It does, however, highlight enduring abstract principles that recur across more than a century of theoretical efforts. Furthermore, it reviews recent formalized frameworks which, although currently heterogeneous and divergent, hint at the possibility of a quantitative groundwork for a future unifying theory.

The field of cognitive neuroscience offers formalized frameworks and general principles designed to track and model the neural correlates of perception, emotion, cognition, and consciousness. These broad frameworks span major levels of description in the brain and attempt to map them onto behavioral and phenomenological data. Corlett et al. (2009, p. 516) argue that until this is done “our understanding of how the pharmacology links to the symptoms will remain incomplete.” Montague et al. (2012, p. 1) argue that ‘computational psychiatry’ can remedy the “lack of appropriate intermediate levels of description that bind ideas articulated at the molecular level to those expressed at the level of descriptive clinical entities.” Seth (2009, p. 50) argues that “computational and theoretical approaches can facilitate a transition from correlation to explanation in consciousness science” and explains how a recent LSD, psilocybin, and ketamine study (Schartner et al., 2017) was motivated by a need to elucidate descriptions at intermediate levels somewhere between pharmacology and phenomenology: “We know there’s a pharmacological link, we know there’s a change in experience and we know there’s a clinical impact. But the middle bit if you like, what are these drugs doing to the global activity of the brain, that’s the gap we’re trying to fill with this study” (quoted in Osborne, 2017). Taken together, the above quotations point to an emerging sense that cognitive neuroscience frameworks can address gaps in our understanding of psychedelic drug effects.

In this article I review theories of psychedelic drug effects. First, making an effort to clearly define the target explananda, I review the acute subjective phenomenological properties of psychedelic effects as well as long-term clinical outcomes from psychedelic-assisted therapies. Second, I review theories from first-wave and second-wave psychedelic science—*model psychoses theory, filtration theory*, and *psychoanalytic theory*—and identify core features of these theories. Third, I review findings from recent neurophysiological research in humans under psychedelic drugs. Finally, I review select 21st-century theories of psychedelic effects that have been developed within cognitive neuroscience frameworks; namely, *entropic brain theory, integrated information theory*, and *predictive processing*. My analysis of recent theoretical efforts highlights certain features, first conceptualized in 19th- and 20th-century theories, which remain relevant in their ability to capture both the phenomenological and neurophysiological dynamics of psychedelic effects. I describe how these enduring theoretical features are now being operationalized into formalized frameworks and could serve as potential unifying principles for describing diverse psychedelic phenomena.

## Psychedelic Drug Effects

**FIGURE 1:**
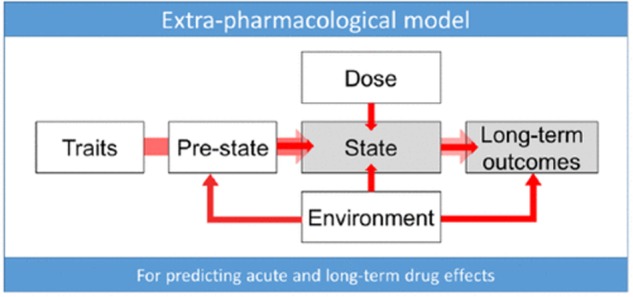
‘Extra-pharmacological’ factors that can determine psychedelic drug effects (Carhart-Harris and Nutt, 2017). “*Trait* factors may be biological [e.g., receptor polymorphisms (Ott, 2007)] or psychological in nature [e.g., personality (MacLean et al., 2011) or suggestibility (Carhart-Harris et al., 2015)]. The *pre-state* refers to such things as anticipatory anxiety, expectations and assumptions (which account for so-called ‘placebo’ and ‘nocebo’ effects), and readiness to surrender resistances and ‘let go’ to the drug effects (e.g., see Russ and Elliott, 2017). In the context of psychedelic research, the pre-state is traditionally referred to as the ‘set’ (Hartogsohn, 2016). *State* refers to the acute subjective and biological quality of the drug experience and may be measured via subjective rating scales or brain imaging (see Roseman et al., 2017). *Dose* relates to the drug dosage—which may be a critical determinant of state (Griffiths et al., 2011; Nour et al., 2016)—as well as long-term outcomes (see Roseman et al., 2017). *Environment* relates to the various environmental influences. In the context of psychedelic research this is traditionally referred to as ‘setting’ (Hartogsohn, 2016). We recognize that the environment can be influential at all stages of the process of change associated with drug action. The *long-term outcomes* may include such things as symptoms of a specific psychiatric condition such as depression—measured using a standard rating scale (Carhart-Harris et al., 2016a) as well as relatively pathology-independent factors such as personality (MacLean et al., 2011) and outlook” (Carhart-Harris and Nutt, 2017, p. 1097).

There are dozens of molecules known to cause psychedelic-like effects (Schultes and Hofmann, 1973; Shulgin and Shulgin, 1991, 1997). This review focuses only on a limited set of drugs dubbed ‘classical hallucinogens’ or ‘classic psychedelics’ which are: LSD, DMT, psilocybin, and mescaline^2^ (Nichols, 2016). Importantly, there are qualitative inter-drug differences between the effects of the four classic psychedelic drugs (Strassman et al., 1994; Hasler et al., 2004; Studerus et al., 2011; Schmid et al., 2015; Liechti et al., 2017). Drug dosage is a primary factor in predicting the types of effects that will occur (Strassman et al., 1994; Riba et al., 2001b; Hasler et al., 2004; Hintzen and Passie, 2010; Studerus et al., 2011, 2012; Liechti et al., 2017). Effects unfold temporally over a drug session; onset effects are distinct from peak effects and some effects have a higher probability of occurring at specific timepoints over the total duration of drug effects (Masters and Houston, 1966; Preller and Vollenweider, 2016). Furthermore, effects are influenced by non-drug factors traditionally referred to as *set and setting*, such as personality, pre-dose mood, drug session environment, and external stimuli (**Figure 1**) (Leary et al., 1963; Studerus et al., 2012; Hartogsohn, 2016; Carhart-Harris and Nutt, 2017).

**FIGURE 2:**
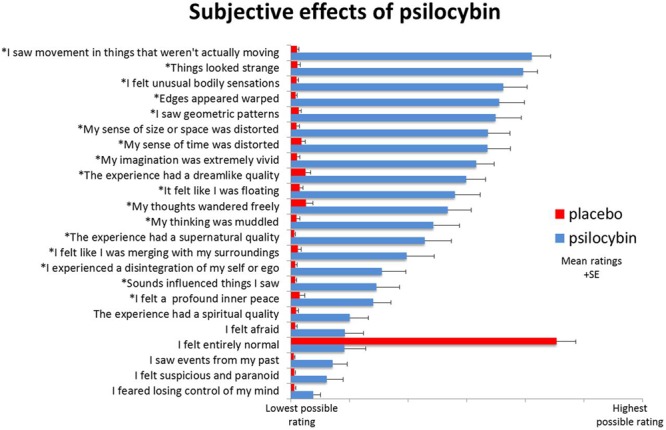
Subjective rating scale items selected after psilocybin (blue) and placebo (red) (*n* = 15) (Muthukumaraswamy et al., 2013). “Items were completed using a visual analog scale format, with a bottom anchor of ‘no, not more than usually’ and a top anchor of ‘yes, much more than usually’ for every item, with the exception of ‘I felt entirely normal,’ which had bottom and top anchors of ‘No, I experienced a different state altogether’ and ‘Yes, I felt just as I normally do,’ respectively. Shown are the mean ratings for 15 participants plus the positive SEMs. All items marked with an asterisk were scored significantly higher after psilocybin than placebo infusion at a Bonferroni-corrected significance level of *p* < 0.0022 (0.5/23 items)” (Muthukumaraswamy et al., 2013, p. 15176).

The above variables, while crucial, do not completely prohibit meaningful characterization of general psychedelic effects, as numerous regularities, patterns, and structure can still be identified (Masters and Houston, 1966; Grinspoon and Bakalar, 1979; Preller and Vollenweider, 2016). Indeed, common psychedelic effects can be reliably measured using validated psychometric instruments consisting of self-report questionnaires and rating scales (Strassman et al., 1994; Dittrich, 1998; Riba et al., 2001a; Dittrich et al., 2010; Studerus et al., 2010, 2011; Maclean et al., 2012; Turton et al., 2014; Barrett et al., 2015; Nour et al., 2016) though some of these rating scales may be in need of further validation using modern statistical techniques (Bouso et al., 2016). Items from these rating scales are wrapped in ‘scare quotes’ in the following discussion in an effort to characterize the subjective phenomenology of psychedelic effects from a first-person perspective. An example of rating scale results is given in (**Figure 2**).

### Perceptual Effects

Perceptual effects occur along a dose-dependent range from subtle to drastic. The range of different perceptual effects includes perceptual intensification, distortion, illusion, mental imagery, elementary hallucination, and complex hallucination (Klüver, 1928; Kometer and Vollenweider, 2016; Preller and Vollenweider, 2016). Intensifications of color saturation, texture definition, contours, light intensity, sound intensity, timbre variation, and other perceptual characteristics are common (Kometer and Vollenweider, 2016; Kaelen et al., 2018). The external world is experienced as if in higher resolution, seemingly more crisp and detailed, often accompanied by a distinct sense of ‘clarity’ or ‘freshness’ in the environment (Hofmann, 1980; Huxley, 1991; Díaz, 2010; Kometer and Vollenweider, 2016). Sense of meaning in percepts is altered, e.g., ‘Things around me had a new strange meaning for me’ or ‘Objects around me engaged me emotionally much more than usual’ (Studerus et al., 2010).

Perceptual distortions and illusions are extremely common, e.g., ‘Things looked strange’ or ‘My sense of size and space was distorted’ or ‘Edges appeared warped’ or ‘I saw movement in things that weren’t actually moving’ (Dittrich, 1998; Muthukumaraswamy et al., 2013). Textures undulate in rhythmic movements, object boundaries warp and pulsate, and the apparent sizes and shapes of objects can shift rapidly (Kometer and Vollenweider, 2016). Controlled psychophysical studies have measured various alterations in motion perception (Carter et al., 2004), object completion (Kometer et al., 2011), and binocular rivalry (Frecska et al., 2004; Carter et al., 2007).

In what are known as *elementary hallucinations*—e.g., ‘I saw geometric patterns’—the visual field can become permeated with intricate tapestries of brightly colored, flowing latticework and other geometric visuospatial ‘form constants’ (Klüver, 1928; Siegel and Jarvik, 1975; Kometer and Vollenweider, 2016). In *complex hallucinations* visual scenes can present elaborate structural motifs, landscapes, cities, galaxies, plants, animals, and human (and non-human) beings (Shanon, 2002; Studerus et al., 2011; Carhart-Harris et al., 2015; Kaelen et al., 2016; Preller and Vollenweider, 2016; Roseman et al., 2016; Kraehenmann et al., 2017b). Complex hallucinations typically succeed elementary hallucinations and are more likely at higher doses (Kometer and Vollenweider, 2016; Liechti et al., 2017) especially under DMT (Strassman et al., 1994; Shanon, 2002). Both elementary and complex hallucinations are more commonly reported behind closed eyelids (‘closed eye visuals’; CEVs) but can dose-dependently occur in full light with eyes open (‘open eye visuals’; OEVs) (Kometer and Vollenweider, 2016). CEVs are often described as vivid mental imagery. Under psychedelic drugs, mental imagery becomes augmented and intensified—e.g., ‘My imagination was extremely vivid’—and is intimately linked with emotional and cognitive effects (Carhart-Harris et al., 2015; Preller and Vollenweider, 2016). “Sometimes sensible film-like scenes appear, but very often the visions consist of scenes quite indescribable in ordinary language, and bearing a close resemblance to the paintings and sculptures of the surrealistic school” (Stockings, 1940, p. 31). Psychedelic mental imagery can be modulated by both verbal (Carhart-Harris et al., 2015) and musical (Kaelen et al., 2016) auditory stimuli. Synaesthesia (Ward, 2013) has been reported, especially visual phenomena driven by auditory stimuli—‘Sounds influenced the things I saw’—but classification of these effects as ‘true’ synaesthesia is actively debated (Sinke et al., 2012; Brogaard, 2013; Luke and Terhune, 2013; Terhune et al., 2016).

Somatosensory perception can be drastically altered—e.g., ‘I felt unusual bodily sensations’—including body image, size, shape, and location (Savage, 1955; Klee, 1963; Preller and Vollenweider, 2016). Sense of time and causal sequence can lose their usual linear cause-effect structure making it difficult to track the transitions between moments (Heimann, 1963; Wittmann et al., 2007; Wackermann et al., 2008; Studerus et al., 2011; Schmid et al., 2015).

Overall the perceptual effects of psychedelics are extremely varied, multimodal, and easily modulated by external stimuli. Perceptual effects are tightly linked with emotional and cognitive effects.

### Emotional Effects

Emotional psychedelic effects are characterized by a general intensification of feelings, increased (conscious) access to emotions, and a broadening in the overall range of emotions felt over the duration of the drug session. Psychedelics can induce unique states of euphoria characterized by involuntary grinning, uncontrollable laughter, silliness, giddiness, playfulness, and exuberance (Preller and Vollenweider, 2016). Negatively experience emotions—e.g., ‘I felt afraid’ or ‘I felt suspicious and paranoid’—are often accompanied by a general sense of losing control, e.g., ‘I feared losing control of my mind’ (Strassman, 1984; Johnson et al., 2008; Barrett et al., 2017a). However, the majority of emotional psychedelic effects in supportive contexts are experienced as positive (Studerus et al., 2011; Schmid et al., 2015; Carhart-Harris et al., 2016b; Belser et al., 2017; Watts et al., 2017). Both LSD and psilocybin can bias emotion toward positive responses to social and environmental stimuli (Kometer et al., 2012; Carhart-Harris et al., 2016b; Dolder et al., 2016; Pokorny et al., 2017). Spontaneous feelings of awe, wonder, bliss, joy, fun, excitement (and yes, peace and love) are also consistent themes across experimental and anecdotal reports (Huxley, 1991; Kaelen et al., 2015; Preller and Vollenweider, 2016; Belser et al., 2017). In supportive environments, classic psychedelic drugs can promote feelings of trust, empathy, bonding, closeness, tenderness, forgiveness, acceptance, and connectedness (Dolder et al., 2016; Belser et al., 2017; Carhart-Harris et al., 2017b; Pokorny et al., 2017; Watts et al., 2017). Emotional effects can be modulated by all types of external stimuli, especially music (Bonny and Pahnke, 1972; Shanon, 2002; Kaelen et al., 2015, 2018).

### Cognitive Effects

Precise characterization of cognitive psychedelic effects has proven enigmatic and paradoxical (Shanon, 2002; Carhart-Harris et al., 2016b). Acute changes in the normal flow of linear thinking—e.g., ‘My thinking was muddled’ or ‘My thoughts wandered freely’—are extremely common (Hasler et al., 2004; Studerus et al., 2011). This is reflected in reduced performance on standardized measures of working memory and directed attention (Carter et al., 2005; Vollenweider et al., 2007); however, reductions in performance have been shown to occur less often in individuals with extensive past experience with the drug’s effects (Bouso et al., 2013). Crucially, cognitive impairments related to acute psychedelic effects are dose-dependent (Wittmann et al., 2007). Extremely low doses, known as *microdoses*, have been anecdotally associated with improvements in cognitive performance (Waldman, 2017; Wong, 2017) “a claim that urgently requires empirical verification through controlled research” (Carhart-Harris and Nutt, 2017, p. 1103). Theoretical attempts to account for the reported effects of microdosing have yet to emerge in the literature and therefore present an important opportunity to future theoretical endeavors.

Certain cognitive traits associated with creativity can increase under psychedelics (Sessa, 2008; Baggott, 2015) such as divergent thinking (Kuypers et al., 2016), use of unlikely language patterns or word associations (Natale et al., 1978b), expansion of semantic activation (Spitzer et al., 1996; Family et al., 2016), and attribution of meaning to perceptual stimuli (Liechti et al., 2017; Preller et al., 2017) especially musical stimuli (Kaelen et al., 2015, 2018; Atasoy et al., 2017b; Barrett et al., 2017b). Primary-process thinking (Rapaport, 1950)—a widely validated psychological construct (Arminjon, 2011) associated with creativity (Suler, 1980)—is characterized phenomenologically by “image fusion; unlikely combinations or events; sudden shifts or transformations of images; and contradictory or illogical actions, feelings, or thoughts” (Kraehenmann et al., 2017a, p. 2). Psilocybin and LSD have been shown to increase primary-process thinking (Martindale and Fischer, 1977; Natale et al., 1978a; Family et al., 2016; Kraehenmann et al., 2017a) as well as the subjective bizarreness and dreamlike nature of mental imagery associated with verbal stimuli (Carhart-Harris et al., 2015; Kraehenmann et al., 2017b). Cognitive flexibility (or ‘loosening’ of cognition) and optimism can remain for up to 2 weeks after the main acute drug effects have dissipated (Carhart-Harris et al., 2016b). Furthermore, long-term increases in creative problem-solving ability (Sweat et al., 2016) and personality trait openness (MacLean et al., 2011; Lebedev et al., 2016) have been measured after just one psychedelic experience.

### Ego Effects and Ego Dissolution Experiences

Klüver (1926, p. 513) observed that under peyote “the line of demarcation drawn between ‘object’ and ‘subject’ in normal state seemed to be changed. The body, the ego, became ‘objective’ in a certain way, and the objects became ‘subjective.”’ Similar observations continued throughout first-wave and second-wave psychedelic science (Beringer, 1927b; Klüver, 1928; Savage, 1955; Eisner and Cohen, 1958; Klee, 1963; Leary et al., 1964; Grof, 1976). Importantly, effects on sense of self and ego occur along a dose-dependent range spanning from subtle to drastic (Letheby and Gerrans, 2017; Millière, 2017). Subtle effects are described as a ‘softening’ of ego with increased insight into one’s own habitual patterns of thought, behavior, personal problems, and past experiences; effects which were utilized in ‘psycholytic’ psychotherapy (Grof, 1980). Drastic ego-effects, known as *ego dissolution^3^*, are described as “the dissolution of the sense of self and the loss of boundaries between self and world” (Millière, 2017, p. 1) —e.g., ‘I felt like I was merging with my surroundings’ or ‘All notion of self and identity dissolved away’ or ‘I lost all sense of ego’ or ‘I experienced a loss of separation from my environment’ or ‘I felt at one with the universe’ (Dittrich et al., 2010; Nour et al., 2016; Millière, 2017). These descriptions resemble non-drug ‘mystical-type’ experiences (James, 1902; Huxley, 1945; Stace, 1960; Forman, 1998; Baumeister and Exline, 2002); however, the extent of overlap here remains an open question (Hood, 2001; Maclean et al., 2012; Barrett and Griffiths, 2017; Millière, 2017; Winkelman, 2017). Ego dissolution is more likely to occur at higher doses (Griffiths et al., 2011; Studerus et al., 2011, 2012; Liechti et al., 2017). Furthermore, certain psychedelic drugs cause ego dissolution experience more reliably than others; psilocybin, for example, was found to produce full ego dissolution more reliably compared with LSD (Liechti et al., 2017). Ego dissolution experiences can be driven and modulated by external stimuli, most notably music (Carhart-Harris et al., 2016c; Atasoy et al., 2017b; Kaelen et al., 2018). Interestingly, subjects who experienced ‘complete’ ego dissolution in psychedelic-assisted therapy were more likely to evidence positive clinical outcomes (Griffiths et al., 2008, 2016; Majić et al., 2015; Ross et al., 2016; Roseman et al., 2017) as well as long-term changes in life outlook and the personality trait openness (MacLean et al., 2011; Carhart-Harris et al., 2016b; Lebedev et al., 2016).

### Clinical Efficacy and Long-Term Effects

Mescaline-assisted therapies showed promising results during first-wave psychedelic science (Beringer, 1927b; Rouhier, 1927) and this trend continued through second-wave psychedelic research on LSD-assisted therapies (Sandison and Whitelaw, 1957; Cohen and Eisner, 1959; Pahnke et al., 1971; Grof, 1976). Recent studies have produced significant evidence for the therapeutic utility of psychedelic drugs in treating a wide range of mental health issues (Tupper et al., 2015; Lieberman and Shalev, 2016; Carhart-Harris and Goodwin, 2017), including anxiety and depression (Grob et al., 2011; Gasser et al., 2014; Carhart-Harris et al., 2016a, 2017a; Dos Santos et al., 2016; Griffiths et al., 2016; Ross et al., 2016), obsessive-compulsive disorder (Moreno et al., 2006), and addiction (Bogenschutz and Johnson, 2016) to alcohol (Bogenschutz et al., 2015) and tobacco (Johnson et al., 2014). In many clinical studies, ego-dissolution experience has correlated with positive clinical outcomes (Griffiths et al., 2008, 2016; Majić et al., 2015; Ross et al., 2016; Roseman et al., 2017).

Remarkably, as mentioned above, a single psychedelic experience can increase optimism for at least 2 weeks after the session (Carhart-Harris et al., 2016b) and can produce lasting changes in personality trait openness (MacLean et al., 2011; Lebedev et al., 2016). A study of regular (weekly) ayahuasca users showed improved cognitive functioning and increased positive personality traits compared with matched controls (Bouso et al., 2015). Interestingly, these outcomes may expand beyond sanctioned clinical use, as illicit users of classic psychedelic drugs within the general population self-report positive long-term benefits from their psychedelic experiences (Carhart-Harris and Nutt, 2010), are statistically less likely to evidence psychological distress and suicidality (Hendricks et al., 2015; Argento et al., 2017), and show an overall lower occurrence of mental health problems in general (Krebs and Johansen, 2013).

### Summary

The above evidence demonstrates the broad diversity of acute subjective effects that classic psychedelic drugs can produce in perceptual, emotional, and cognitive domains. Unique changes in sense of self, ego, body image, and personal meaning are particularly salient themes. How do these molecules produce such dramatic effects? What are the relationships between acute perceptual, emotional, cognitive, and self-related effects? What is the link between acute effects and long-term changes in mental health, personality, and behavior? Theories addressing these questions emerged as soon as Western science recognized the need for a scientific understanding of psychedelic drug effects beginning in the late 19th century.

## 19th and 20th Century Theories of Psychedelic Drug Effects

The effects described above are what captured the interest of first-wave and second-wave psychedelic scientists, and the theories they developed in their investigations have two central themes. The first theme is the observation that psychedelic effects share descriptive elements with symptoms of psychoses, such as hallucination, altered self-reference, and perceptual distortions. This theme forms the basis of *model psychoses theory* and is what motivated the adoption of the term ‘psychotomimetic’ drugs. The second theme is the observation that psychedelic drugs seem to expand the total range of contents presented subjectively in our perceptual, emotional, cognitive, and self-referential experience. This theme forms the basis of *filtration theory* and is what motivated the adoption of the term ‘psychedelic’ drugs. A third theoretical account uses *psychoanalytic theory* to address the expanded range of mental phenomena produced by psychedelic drugs as well as the shared descriptive elements with symptoms of psychoses. The next section reviews these themes along with their historically associated theories before tracing their evolution into third-wave (21st-century) psychedelic science.

### Model Psychoses Theory

When Lewin (1894, 1927) ‘discovered’^4^ the peyote cactus, his reports caught the attention of adventurous 19th-century scientists like Prentiss and Morgan (1895), Mitchell (1896), and Ellis (1898), who promptly obtained samples and began consuming the cactus and observing its effects on themselves. When Heffter (1898) isolated mescaline from the peyote cactus and Späth (1919) paved the way for laboratory synthesis, scientists began systematically dosing themselves (along with their colleagues and students) with mescaline and publishing their findings in medical journals (Knauer and Maloney, 1913; Klüver, 1926; Beringer, 1927b; Rouhier, 1927; Guttmann, 1936; Stockings, 1940). Klüver (1926), intrigued by the approach of Knauer and Maloney (1913), ingested peyote at the University of Minnesota Psychological Laboratory and, after the effects had taken hold, completed standard psychophysical measures. Klüver (1926, p. 502) argued that systematic investigations into the neural mechanisms of mescaline effects would help neurology “elucidate more general questions of the psychology and pathology of perception.” However, it was the pathology aspect, not the general psychology questions, which became the dominant focus of ensuing mescaline research paradigms.

Model psychoses theory began long before any of the classic psychedelic drugs became known to Western science. Moreau (1845) linked hashish effects with mental illness and Kraepelin (1892) founded “pharmacopsychology” by dosing himself and his students with various psychoactive drugs in the laboratory of Wilhelm Wundt (Müller et al., 2006; Schmied et al., 2006). These scientists hoped to study psychotic symptoms using drugs to induce ‘model psychoses’ (1) in themselves, to gain first-person knowledge of the phenomenology of psychotic symptoms by “administering to one another such substances as will produce in us transitory psychoses” (Knauer and Maloney, 1913, p. 426; see also Guttmann, 1936), and (2) in normal research subjects, allowing for laboratory behavioral observations on how the symptoms emerge and dissipate. Kraepelin and colleagues attempted to model psychoses using many drugs—“tea, alcohol, morphine, trional, bromide, and other drugs”—yet Kraepelin’s pupils Knauer and Maloney (1913, p. 426) argued that these drugs unfortunately “produce mental states which have little similarities to actual insanities” and argued instead that *mescaline* was unique in its ability to truly model psychoses. The dramatic subjective effects of mescaline invigorated the model psychoses paradigm. Growing demand for the ideal chemical agent for model psychoses eventually motivated Sandoz Pharmaceuticals to bring LSD to market in the 1940s.^5^

Importantly, model psychoses theory was not initially a theory of drug effects; it was an idealistic paradigm for researching psychoses that was already in use before Western science ‘discovered’ classic psychedelic drugs. Nonetheless, it seeded the idea that psychedelic effects themselves could be explained in terms of psychopathology and motivated a search for common neural correlates. The founding figures of neuropharmacology were driven by questions regarding the relationship between psychoactive drugs and endogenous neurochemicals (see Abramson, 1956). The putative psychoses-mimicking effects of LSD and mescaline inspired the idea that psychotic symptoms might be caused by a “hypothetical endotoxin” (Osmond, 1957, p. 422) or some yet-unknown endogenous neurochemical gone out of balance (Osmond and Smythies, 1952; Abramson, 1956; Himwich, 1959). The discovery that LSD can antagonize serotonin led to the hypothesis that the effects of LSD are serotonergic and simultaneously to the historic hypothesis^6^ that serotonin might play a role in regulating mental function (Gaddum, 1953; Gaddum and Hameed, 1954; Woolley and Shaw, 1954; Shaw and Woolley, 1956; Green, 2008).

At the 1955 *Second Conference on Neuropharmacology* the whole class of drugs was dubbed *psychotomimetic* (Abramson, 1956). Interestingly, the word *mimetic* means to “imitate” “mimic” or “exhibit mimicry” which is the act of *appearing* as something else—for example, when one species mimics the appearance or behavior of another (e.g., the non-venomous bullsnake rattles its tail against dry leaves to *mimic* a venomous rattlesnake). Psychoto*mimetic* drug effects, on this literal reading of the term, would merely mimic or imitate—appear as if they are—psychoses. However, to mimic is not to model.^7^ A model intends to capture important structural or functional principles of the entity or phenomena that it models. A mimic, by contrast, merely creates the illusion that it possesses the properties it mimics. Thus, the term *psychotomimetic* implies that the effects of these drugs merely resemble psychoses but do not share functional or structural properties in their underlying biology or phenomenology. Nonetheless, LSD and mescaline were used as *models* to investigate psychotic symptoms. Yet the scientific utility of drug models hinges on our understanding of the mechanisms underpinning the drugs’ effects; we still need a theory of how psychotomimetic drugs work. A subtle explanation-explananda circularity can come into play here, in which psychoses are explained using drug models yet the drug effects are explained using theories of psychoses. Further complicating the matter is the clear difference between *acutely induced* drug effects and the gradual development of a *chronic* mental illness (Osmond and Smythies, 1952). This cluster of conceptual challenges poured fuel on the flaming debates about the merits of drug-induced model psychoses, which in 1957 had already “smoldered for nearly 50 years” (Osmond, 1957, p. 421). An additional conceptual challenge was the fact that mescaline had for years shown promise in *treating* psychopathologies (Beringer, 1927b; Rouhier, 1927) and LSD was gaining popularity for pharmaceutically enhanced psychotherapy (Sandison and Whitelaw, 1957; Eisner and Cohen, 1958; Cohen and Eisner, 1959). Model psychoses theory needed to explain how it was the case that drugs putatively capable of inducing psychotic symptoms could simultaneously be capable of treating them—What Osmond (1957, p. 420) termed the “hair of the dog” problem. In fact, to this day “the apparent paradox by which the same compound can be both a model of, and yet a treatment for, psychopathology has never been properly addressed” (Carhart-Harris et al., 2016b, p. 2). Taken together, the above cluster of conceptual challenges drove Osmond (1957) to doubt his own prior work on model psychoses (Hoffer et al., 1954; i.e., Osmond and Smythies, 1952) and he declared ‘psychotomimetic’ an outmoded term, arguing that the effects of these drugs could not be captured wholly in terms of psychopathology. “If mimicking mental illness were the main characteristic of these agents, ‘psychotomimetics’ would indeed be a suitable generic term. It is true that they do so, but they do much more” (Osmond, 1957, p. 429).

### Filtration Theory

Osmond (1957) argued that the ‘psychotomimetic’ class of drugs needed a more appropriate name. “My choice, because it is clear, euphonious, and uncontaminated by other associations, is *psychedelic*, mind-manifesting” (Osmond, 1957, p. 429). But how exactly should we understand psychedelic effects as ‘mind-manifesting’? Osmond’s nomenclature legacy was directly influenced by his friend Aldous Huxley, who described the core idea to Osmond in the following personal letter dated April 10, 1953 (Huxley, 1953, p. 29):

Dear Dr. Osmond,

…

It looks as though the most satisfactory working hypothesis about the human mind must follow, to some extent, the Bergsonian model, in which the brain with its associated normal self, acts as a utilitarian device for limiting, and making selections from, the enormous possible world of consciousness, and for canalizing experience into biologically profitable channels. Disease, mescaline, emotional shock, aesthetic experience and mystical enlightenment have the power, each in its different way and in varying degrees, to inhibit the function of the normal self and its ordinary brain activity, thus permitting the ‘other world’ to rise into consciousness.

*Yours sincerely*,

Aldous Huxley

**FIGURE 3:**
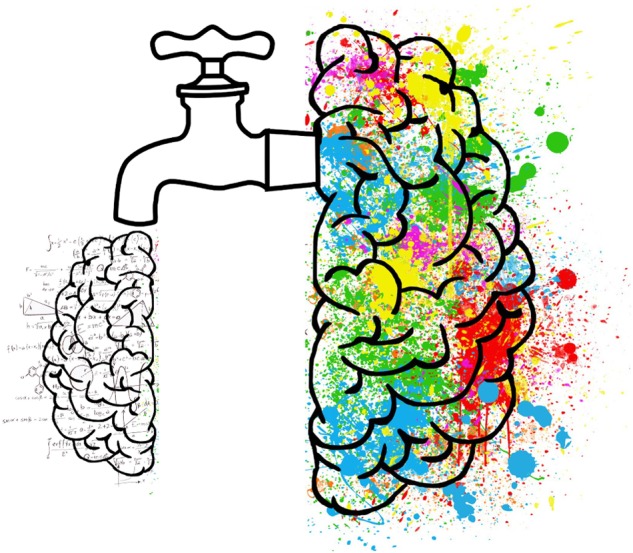
Aldous Huxley’s “cerebral reducing valve.” On the ‘inlet’ (right) side of the cerebral reducing valve is a vast ocean of all possible perceptual, emotional, and cognitive experiences. On the ‘outlet’ (left) side is our moment-to-moment stream of experience in normal waking life. Mechanisms inside the valve ‘reduce’ the character and contents of experience, ‘canalizing’ the ocean of possible experience into a more limited stream of waking consciousness aimed at maximum biological utility.

Huxley’s letter can help unpack the intended ‘mind-manifesting’ etymology of Osmond’s new term *psychedelic*. Huxley saw the biological function of the brain as a “device” engaged in a continuous process of *elimination and inhibition* to sustain the “normal self” of everyday waking experience to maximize adaptive fit. Huxley’s choice metaphor for visualizing this was the *cerebral reducing valve* (**Figure 3**).

“What I have called the cerebral reducing valve [is a] normal brain function that limits our mental processes to an awareness, most of the time, of what is biologically useful” (Huxley, 1999, p. 121). Huxley (1961b, p. 193) argued that this “normal brain function” emerges developmentally during the course of psychological maturity, so for a period during childhood, before the cerebral reducing valve has fully developed, “there is this capacity to live in a kind of visionary world.” Once the valve is fully developed, however, normal waking life becomes restricted to a “world fabricated by our everyday, biologically useful and socially conditioned perceptions, thoughts and feelings” (Huxley, 1961a, p. 214).

Huxley borrowed the core idea from 19th-century *filtration theory* accounts of various mental phenomena (see Marshall, 2005): “According to filtration theorists, consciousness is ordinarily kept narrow by biological and psychological selection processes that exclude a great deal of subconscious material” (Marshall, 2005, p. 233). Filtration theorists include founding figures of psychopharmacology (Kraepelin, 1892), psychology (James, 1890), and parapsychology (Myers, 1903), along with early 20th-century philosophers Bergson (1911, 1931) and Broad (1923). Bergson (1931) applied his own filtration framework to drug effects in his brief response to James’ (1882) glowing descriptions of what it is like to inhale nitrous oxide. James’ peculiar state of mind, explained Bergson, should be thought of as a latent potential of the brain/mind, which nitrous oxide simply “brought about materially, by an inhibition of what inhibited it, by the removing of an obstacle; and this effect was the wholly negative one produced by the drug” (Bergson, 1931). Huxley picked up Bergson’s line of thinking and eventually convinced Osmond that it was important to reflect this principle in scientific descriptions of the effects of LSD and mescaline. Smythies (1956, p. 96) also subscribed to this idea, stating that “mescaline may be supposed to inhibit that function in the brain which specifically inhibits the mescaline phenomena from developing in the sensory fields.”

Thus, Osmond’s (1957) proposed name-change—*psychedelic*—was intended to capture the spirit of filtration theory. In this new descriptive model, *psyche* (mind) *delic* (manifesting) drugs manifest the mind by inhibiting certain brain processes which normally maintain their own inhibitory constraints on our perceptions, emotions, thoughts, and sense of self. Osmond (1957) and Huxley (1991) both found this principle highly applicable to their own direct first-person knowledge of what it is like to experience the effects of mescaline and LSD—the expanded range of feelings, intensification of perceptual stimuli, vivid vision-like mental imagery, unusual thoughts, and expanding (or dissolving) sense of self and identity.

Osmond argued that his ‘mind-manifesting’ description had further theoretical virtues that could address the conceptual challenges of model psychoses theory and improve our understanding of (1) the diverse range of psychedelic effects, (2) their relationship to psychotic symptoms, and (3) their role in psychedelic-assisted therapies. First, the pharmacological disruption of hypothetical inhibitory brain mechanisms that normally attenuate internal and external stimuli suggested that the kinds of effects produced by the drug would depend on the kinds of stimuli in the system, which is consistent with the diverse range of effects on multiple perceptual modalities, emotional experience, and cognition.

Second, the brain’s selective filtration mechanisms, while evolutionarily adaptive and biologically useful, could develop pathological characteristics in two fundamentally distinct ways. First, a chronically *overactive* filter limits too much of the mind, causing a rigid, dull, neurotic life in which mental contents become overly restricted to “those enumerated in the Sears-Roebuck catalog which constitutes the conventionally ‘real’ world” (Huxley, 1953, p. 30). Second, a chronically *underactive* or ‘leaky’ filter places too few constraints on the mind and allows too much ‘Mind at Large’ to enter conscious awareness, potentially resulting in perceptual instability, cognitive confusion, or hallucination. This picture helped Huxley and Osmond understand the relationship between psychedelic phenomena and psychotic phenomena: temporarily opening the cerebral reducing valve with psychedelics could produce mental phenomena that resembled symptoms of chronic natural psychoses precisely because both were the result of (acute or chronic) reductions in brain filtration mechanisms.

Third and finally, filtration theory addressed the paradoxical “hair of the dog” issue—why drugs that ‘mimic’ psychoses can aid psychotherapy—which, as described in the previous section, was a conceptual challenge for model psychoses theory. The solution to the paradox was in the filtration theory idea that psychedelic drugs temporarily ‘disable’ brain filtration mechanisms, which could allow patients and therapists to work outside of the patient’s everyday (pathological) inhibitory mechanisms. Thus, filtration theory offered a way to understand psychedelic effects that was consistent with both their psychotomimetic properties and their therapeutic utility.

Osmond and Huxley argued that filtration theory concepts were fully consistent with the subjective phenomenology, psychotomimetic capability, and therapeutic efficacy of psychedelic drugs. However, it remains unclear exactly *what it is* that the brain is filtering and consequently *what it is* that emerges when the filter is pharmacologically perturbed by a psychedelic drug. According to Huxley, LSD and mescaline “inhibit the function of the normal self and its ordinary brain activity, thus permitting the ‘*other world*’ to rise into consciousness” (Huxley, 1953, p. 29; emphasis mine). Huxley (and Bergson) spoke of the brain as a device that filters the *world* and when the filter is removed we experience ‘more’ of reality. Osmond’s ‘mind-manifesting’ (*psyche*) (*delic*) name, by contrast, suggests that these drugs permit latent aspects of *mind* to rise into conscious awareness. So which is it? Do psychedelic drugs manifest latent aspects of *mind* or of *world*? How we answer this question will crucially determine our ontological and epistemological conclusions regarding the nature of psychedelic experience. Huxley and Osmond did not make this clear. Huxley seems to favor the position that psychedelic experience reveals a wider ontological reality and grants epistemic access to greater truth. Osmond’s view, on which these drugs reveal normally hidden aspects of mind, seems less radical, more compatible with materialist science, and less epistemically and ontologically committed. Still, if mind provides us with access to world, then lifting restrictions on mind could in principle expand our access to world. This important point resurfaces in section “Predictive Processing” below.

### Psychoanalytic Theory

Freud (1895) developed an elaborate theoretical account of mental phenomena which, like filtration theory, placed great emphasis on inhibition mechanisms in the nervous system.^8^ Freud divided the psyche into two fundamentally distinct modes of activity: *the primary process* and *the secondary process* (Freud, 1895, 1940). In the primary process, the exchange of “neuronal energy” is “freely mobile” and its psychological dynamics are characterized by disorder, vagueness, conceptual paradox, symbolic imagery, intense emotions, and animistic thinking (Freud, 1940, p. 164). In the secondary process, by contrast, the exchange of neuronal energy is “bound” and its psychological dynamics are characterized by order, precision, conceptual consistency, controlled emotions, and rational thinking (Freud, 1895, 1940). Freud (1895) hypothesized that the secondary process is maintained by an organizing neural “mass” called the ego which “contains” and exerts control over the primary process by binding primary process activity into its own pattern of activity.^9^ Freud hypothesized that secondary process neural organization, sustained by the ego, is required for certain aspects of perceptual processing, directed attention, reality-testing, sense of linear time, and higher cognitive processes (Freud, 1895, 1940). When Freud’s ego is suppressed, such as during dream sleep, wider worlds of experience can emerge, but secondary process functions are lost. The secondary process and its supporting neural organizing pattern—the ego—emerges during ontogenetic development and solidifies with adult maturity: “A unity comparable to the ego cannot exist from the start; the ego has to be developed” (Freud, 1915, p. 77). Furthermore, pathological characteristics can emerge when Freud’s ego restricts either *too much* or *too little* of the primary process.

Freud himself was apparently uninterested in psychedelic drugs and instead emphasized dreams as “the royal road to a knowledge of the unconscious activities of the mind” (Freud, 1900, p. 769). Nonetheless, psychedelic drugs produce dream*like* visions and modes of cognition that feature symbolic imagery, conceptual paradox, and other hallmark characteristics of the primary process (Carhart-Harris and Friston, 2010; Kraehenmann et al., 2017a; Sanz and Tagliazucchi, 2018). How did other psychoanalytic theorists describe psychedelic drug effects? The core idea is that psychedelic drugs interfere with the structural integrity of the ego and thereby reduce its ability to suppress the primary process and support the secondary process (Grof, 1976). This ‘frees’ the primary process which then spills into conscious awareness, resulting in perceptual instability, wildly vivid imagination, emotional intensity, conceptual paradox, and loss of usual self-boundaries. Due in part to the close resemblance between psychedelic effects and primary process phenomena, psychoanalytic theory became the framework of choice during the mid 20th-century boom in psychedelic therapy (Sandison, 1954; Sandison and Whitelaw, 1957; Cohen, 1965; Grof, 1976; Merkur, 1998). Psychedelic ego effects, which range from a subtle loosening to a complete dissolution of ego boundaries, were found to be great tools in psychotherapy because of their capacity to perturb ego and allow primary process phenomena to emerge (Sandison, 1954, p. 509).

But *how* do psychedelic drugs disrupt the structure of the ego? Freud hypothesized that the organizational structure of ego rests upon a basic perceptual schematic of the body and its surrounding environment. Perceptual signals are continuously ‘bound’ and integrated into the somatic boundaries of the ego. Savage (1955) speculated that the LSD’s perceptual effects and ego effects are tightly linked. “LSD acts by altering perception. Continuous correct perception is necessary to maintain ego feeling and ego boundaries. … *Perception determines our ego boundaries.* … disturbances in perception caused by LSD make it impossible for the ego to integrate the evidence of the senses and to coordinate its activities …” (Savage, 1955, p. 14). Klee (1963) expanded Savage’s insights into a set of hypotheses aimed at elucidating the neurobiological mechanisms of a Freudian ‘stimulus barrier’ and its dissolution under LSD:

Such barriers would presumably consist of processes limiting the spread of excitation between different functional areas of the brain. The indications are that LSD, in some manner, breaks down these stimulus barriers of which Freud spoke. *Nor is this merely a figure of speech*. There is some reason to suspect that integrative mechanisms within the central nervous system (CNS) which handle inflowing stimuli are no longer able to limit the spread of excitation in the usual ways. We might speculate that LSD allows greater energy exchanges between certain systems than normally occurs, without necessarily raising the general level of excitation of all cortical and subcortical structures. (Klee, 1963, p. 465; emphasis mine).

Freud hypothesized that ego is sustained by a delicate balance of ‘neuronal energy’ which critically depends on integrative mechanisms to process inflowing sensory stimuli and to ‘bind’ neural excitation into functional structures within the brain. Psychedelic drugs, according to Savage and Klee, perturb integrative mechanisms that normally bind and shape endogenous and exogenous excitation into the structure of the ego. As we will see below, Klee’s ideas strongly anticipate many neurophysiological findings (Alonso et al., 2015; Tagliazucchi et al., 2016; Schartner et al., 2017) and theoretical themes (Carhart-Harris and Friston, 2010; Letheby and Gerrans, 2017) from 21st-century psychedelic science.

### Summary

From the above analysis of first-wave and second-wave theories I have identified four recurring theoretical features which could potentially serve as unifying principles. One feature is the hypothesis that psychedelic drugs inhibit a core brain mechanism that normally functions to ‘reduce’ or ‘filter’ or ‘constrain’ mental phenomena into an evolutionarily adaptive container. A second feature is the hypothesis that this core brain mechanism can behave pathologically, either in the direction of too much, or too little, constraint imposed on perception, emotion, cognition, and sense of self. A third feature is the hypothesis that psychedelic phenomena and symptoms of chronic psychoses share descriptive elements because they both involve situations of relatively *unconstrained* mental processes. A fourth feature is the hypothesis that psychedelic drugs have therapeutic utility via their ability to temporarily inhibit these inhibitory brain mechanisms. But how are these inhibitory mechanisms realized in the brain?

## Neuropharmacology and Neurophysiological Correlates of Psychedelic Drug Effects

Klee recognized that his above hypotheses, inspired by psychoanalytic theory and LSD effects, required neurophysiological evidence. “As far as I am aware, however, adequate neurophysiological evidence is lacking … The long awaited millennium in which biochemical, physiological, and psychological processes can be freely correlated still seems a great distance off” (Klee, 1963, p. 466, 473). What clues have recent investigations uncovered?

A psychedelic drug molecule impacts a neuron by binding to and altering the conformation of receptors on the surface of the neuron (Nichols, 2016). The receptor interaction most implicated in producing classic psychedelic drug effects is agonist or partial agonist activity at serotonin (5-HT) receptor type 2A (5-HT_2A_) (Nichols, 2016). A molecule’s propensity for 5-HT_2A_ affinity and agonist activity predicts its potential for (and potency of) subjective psychedelic effects (Glennon et al., 1984; McKenna et al., 1990; Halberstadt, 2015; Nichols, 2016; Rickli et al., 2016). When a psychedelic drug’s 5-HT_2A_ agonist activity is intentionally blocked using 5-HT_2A_ *antagonist* drugs (e.g., ketanserin), the subjective effects are blocked or attenuated in humans under psilocybin (Vollenweider et al., 1998; Kometer et al., 2013), LSD (Kraehenmann et al., 2017a,b; Preller et al., 2017), and ayahuasca (Valle et al., 2016). Importantly, while the above evidence makes it clear that 5-HT_2A_ activation is a necessary (if not sufficient) mediator of the hallmark subjective effects of classic psychedelic drugs, this does not entail that 5-HT_2A_ activation is the sole neurochemical cause of all subjective effects. For example, 5-HT_2A_ activation might trigger neurochemical modulations ‘downstream’ (e.g., changes in glutamate transmission) which could also play causal roles in producing psychedelic effects (Nichols, 2016). Moreover, most psychedelic drug molecules activate other receptors in addition to 5-HT_2A_ (e.g., 5-HT_1A_, 5-HT_2C_, dopamine, sigma, etc.) and these activations may importantly contribute to the overall profile of subjective effects even if 5-HT_2A_ activation is required for their effects to occur (Ray, 2010, 2016).

How does psychedelic drug-induced 5-HT_2A_ receptor agonism change the behavior of the host neuron? Generally, 5-HT_2A_ activation has a depolarizing effect on the neuron, making it more excitable (more likely to fire) (Andrade, 2011; Nichols, 2016). Importantly, this does not necessarily entail that 5-HT_2A_ activation will have an overall excitatory effect throughout the brain, particularly if the excitation occurs in inhibitory neurons (Andrade, 2011). This important consideration (captured by the adage ‘one neuron’s excitation is another neuron’s inhibition’) should be kept in mind when tracing causal links in the pharmaco-neurophysiology of psychedelic drug effects.

In mammalian brains, neurons tend to ‘fire together’ in synchronized rhythms known as *temporal oscillations* (brain waves). MEG and EEG equipment measure the electromagnetic disturbances produced by the temporal oscillations of large neural populations and these measurements can be quantified according to their *amplitude* (power) and *frequency* (timing) (Buzsáki and Draguhn, 2004). Specific combinations of frequency and amplitude can be correlated with distinct brain states, including waking ‘resting’ state, various attentional tasks, anesthesia, REM sleep, and deep sleep (Tononi and Koch, 2008; Atasoy et al., 2017a). In what ways do temporal oscillations change under psychedelic drugs? MEG and EEG studies consistently show *reductions* in oscillatory power across a broad frequency range under ayahuasca (Riba et al., 2002, 2004; Schenberg et al., 2015; Valle et al., 2016), psilocybin (Muthukumaraswamy et al., 2013; Kometer et al., 2015; Schartner et al., 2017), and LSD (Carhart-Harris et al., 2016c; Schartner et al., 2017). Reductions in the power of alpha-band oscillations, localized mainly to parietal and occipital cortex, have been correlated with intensity of subjective visual effects—e.g., ‘I saw geometric patterns’ or ‘My imagination was extremely vivid’—under psilocybin (Kometer et al., 2013; Muthukumaraswamy et al., 2013; Schartner et al., 2017) and ayahuasca (Riba et al., 2004; Valle et al., 2016). Under LSD, reductions in alpha power still correlated with intensity of subjective visual effects but associated alpha reductions were more widely distributed throughout the brain (Carhart-Harris et al., 2016c). Furthermore, ego-dissolution effects and mystical-type experiences (e.g., ‘I experienced a disintegration of my “self” or “ego”’ or ‘The experience had a supernatural quality’) have been correlated with reductions in alpha power localized to anterior and posterior cingulate cortices and the parahippocampal regions under psilocybin (Muthukumaraswamy et al., 2013; Kometer et al., 2015) and throughout the brain under LSD (Carhart-Harris et al., 2016c).

The concept of *functional connectivity* rests upon fMRI brain imaging observations that reveal temporal correlations of activity occurring in spatially remote regions of the brain which form highly structured patterns (brain networks) (Buckner et al., 2013). Imaging of brains during perceptual or cognitive task performance reveals patterns of functional connectivity known as *functional networks*; e.g., control network, dorsal attention network, ventral attention network, visual network, auditory network, and so on. Imaging brains in taskless resting conditions reveals *resting-state functional connectivity* (RSFC) and structured patterns of RSFC known as resting state networks (RSNs; Deco et al., 2011). One particular RSN, the default mode network (DMN; Buckner et al., 2008), increases activity in the absence of tasks and decreases activity during task performance (Fox and Raichle, 2007). DMN activity is strong during internally directed cognition and a variety of other ‘metacognitive’ functions (Buckner et al., 2008). DMN activation in normal waking states exhibits ‘inverse coupling’ or anticorrelation with the activation of task-positive functional networks, meaning that DMN and functional networks are often mutually exclusive; one deactivates as the other activates and vice versa (Fox and Raichle, 2007).

In what ways does brain network connectivity change under psychedelic drugs? First, functional connectivity between key ‘hub’ areas—mPFC and PCC—is reduced. Second, the ‘strength’ or oscillatory power of the DMN is weakened and its intrinsic functional connectivity becomes disintegrated as its component nodes become decoupled under psilocybin (Carhart-Harris et al., 2012, 2013), ayahuasca (Palhano-Fontes et al., 2015), and LSD (Carhart-Harris et al., 2016c; Speth et al., 2016). Third, brain networks that normally show anticorrelation become active simultaneously under psychedelic drugs. This situation, which can be described as increased *between-network* functional connectivity, occurs under psilocybin (Carhart-Harris et al., 2012, 2013; Roseman et al., 2014; Tagliazucchi et al., 2014), ayahuasca (Palhano-Fontes et al., 2015) and especially LSD (Carhart-Harris et al., 2016c; Tagliazucchi et al., 2016). Fourth and finally, the overall repertoire of explored functional connectivity motifs is substantially expanded and its informational dynamics become more diverse and entropic compared with normal waking states (Tagliazucchi et al., 2014, 2016; Alonso et al., 2015; Lebedev et al., 2016; Viol et al., 2016; Atasoy et al., 2017b; Schartner et al., 2017). Notably, the magnitude of occurrence of the above four neurodynamical themes correlates with subjective intensity of psychedelic effects during the drug session. Furthermore, visual cortex is activated during eyes-closed psychedelic visual imagery (de Araujo et al., 2012; Carhart-Harris et al., 2016c) and under LSD “the early visual system behaves ‘as if’ it were receiving spatially localized visual information” as V1-V3 RSFC is activated in a retinotopic fashion (Roseman et al., 2016, p. 3036).

Taken together, the recently discovered neurophysiological correlates of subjective psychedelic effects present an important puzzle for 21st-century neuroscience. A key clue is that 5-HT_2A_ receptor agonism leads to desynchronization of oscillatory activity, disintegration of intrinsic integrity in the DMN and related brain networks, and an overall brain dynamic characterized by increased between-network global functional connectivity, expanded signal diversity, and a larger repertoire of structured neurophysiological activation patterns. Crucially, these characteristic traits of psychedelic brain activity have been correlated with the phenomenological dynamics and intensity of subjective psychedelic effects.

## 21st-Century Theories of Psychedelic Drug Effects

How should we understand the growing body of clues emerging from investigations into the neurodynamics of psychedelic effects? What are the principles that link these thematic patterns of psychedelic brain activity (or inactivity) to their associated phenomenological effects? Recent theoretical efforts to understand psychedelic drug effects have taken advantage of existing frameworks from cognitive neuroscience designed to track the key neurodynamic principles of human perception, emotion, cognition, and consciousness. The overall picture that emerges from these efforts shares core principles with filtration and psychoanalytic accounts of the late 19th and early 20th century. Briefly, normal waking perception and cognition are hypothesized to rest upon brain mechanisms which serve to suppress entropy and uncertainty by placing various *constraints* on perceptual and cognitive systems. In a ‘selecting’ and ‘limiting’ fashion, neurobiological constraint mechanisms support stability and predictability in the contents of conscious awareness in the interest of adaptability, survival, and evolutionary fitness. The core hypothesis of recent cognitive neuroscience theories of psychedelic effects is that these drugs interfere with the integrity of neurobiological information-processing constraint mechanisms. The net effect of this is that the range of possibilities in perception, emotion, and cognition is dose-dependently expanded. From this core hypothesis, cognitive neuroscience frameworks are utilized to describe and operationalize the quantitative neurodynamics of key psychedelic phenomena; namely, the diversity of effects across many mental processes, the elements in common with symptoms of psychoses, and the way in which temporarily removing neurobiological constraints is therapeutically beneficial.

This section is organized according to the broad theoretical frameworks informing recent theoretical neuroscience of psychedelic effects: *entropic brain theory, integrated information theory*, and *predictive processing*.

### Entropic Brain Theory

Entropic Brain Theory (EBT; Carhart-Harris et al., 2014) links the phenomenology and neurophysiology of psychedelic effects by characterizing both in terms of the quantitative notions of entropy and uncertainty. Entropy is a quantitative index of a system’s (physical) disorder or randomness which can simultaneously describe its (informational) uncertainty. EBT “proposes that the quality of any conscious state depends on the system’s entropy measured via key parameters of brain function” (Carhart-Harris et al., 2014, p. 1). Their hypothesis states that hallmark psychedelic effects (e.g., perceptual destabilization, cognitive flexibility, ego dissolution) can be mapped directly onto elevated levels of entropy/uncertainty measured in brain activity, e.g., widened repertoire of functional connectivity patterns, reduced anticorrelation of brain networks, and desynchronization of RSN activity. More specifically, EBT characterizes the difference between psychedelic states and normal waking states in terms of how the underlying brain dynamics are positioned on a scale between the two extremes of order and disorder—a concept known as ‘self-organized criticality’ (Beggs and Plenz, 2003). A system with high order (low entropy) exhibits dynamics that resemble ‘petrification’ and are relatively inflexible but more stable, while a system with low order (high entropy) exhibits dynamics that resemble ‘formlessness’ and are more flexible but less stable. The notion of ‘criticality’ describes the transition zone in which the brain remains poised between order and disorder. Physical systems at criticality exhibit increased transient ‘metastable’ states, increased sensitivity to perturbation, and increased propensity for cascading ‘avalanches’ of metastable activity. Importantly, EBT points out that these characteristics are consistent with psychedelic phenomenology, e.g., hypersensitivity to external stimuli, broadened range of experiences, or rapidly shifting perceptual and mental contents. Furthermore, EBT uses the notion of criticality to characterize the difference between psychedelic states and normal waking states as it “describes cognition in adult modern humans as ‘near critical’ but ‘sub-critical’—meaning that its dynamics are poised in a position between the two extremes of formlessness and petrification where there is an optimal balance between order and flexibility” (Carhart-Harris et al., 2014, p. 12). EBT hypothesizes that psychedelic drugs interfere with ‘entropy-suppression’ brain mechanisms which normally sustain sub-critical brain dynamics, thus bringing the brain “closer to criticality in the psychedelic state” (Carhart-Harris et al., 2014, p. 12).

Entropic Brain Theory further characterizes psychedelic neurodynamics using a neo-psychoanalytic framework proposed in an earlier paper by Carhart-Harris and Friston (2010, p. 1265) where they “recast some central Freudian ideas in a mechanistic and biologically informed fashion.” Freud’s primary process (renamed “primary consciousness”) is hypothesized to be a high-entropy brain dynamic which operates at criticality, while Freud’s secondary process (renamed “secondary consciousness”) is hypothesized to involve a lower-entropy brain state which sustains a sub-critical dynamic via a key neurobiological entropy-suppression mechanism—the ego—which exerts an organizing influence in order to constrain the criticality-like dynamic of primary consciousness. EBT argues that these ego functions have a signature neural footprint; namely, the DMN’s intrinsic functional connectivity and DMN coupling of medial temporal lobes (MTLs) in particular. Furthermore, EBT argues that DMN/ego develops ontogenetically in adult humans and plays an adaptive role in which it sustains secondary consciousness and associated metacognitive abilities (Shimamura, 2000; Fleming et al., 2012) along with an “integrated sense of self” (Carhart-Harris et al., 2014, p. 9).

Importantly, this hypothesis maps onto the subjective phenomenology of psychedelic effects, particularly ego dissolution. As psychedelics weaken the oscillatory power and intrinsic functional connectivity of the DMN, the normally constrained activity of subordinate DMN nodes—MTLs in particular—becomes “freely mobile” allowing the emergence of more uncertain (higher entropy) primary consciousness. This view, based on Freudian metapsychology, is also consistent with filtration accounts, like those of Bergson and Huxley, who hypothesized that psychedelic drug effects are the result of a pharmacological *inhibition* of inhibitory brain mechanisms. EBT recasts these theoretical features using the quantitative terms of physical entropy and informational uncertainty as measured via “the repertoire of functional connectivity motifs that form and fragment across time” (Carhart-Harris et al., 2014, p. 1). In normal waking states, the DMN *constrains* the activity of its cortical and subcortical nodes and prohibits simultaneous co-activation with TPNs. By interfering with DMN integration, psychedelics permit a larger repertoire of brain activity, a wider variety of explored functional connectivity motifs, co-activation of normally mutually exclusive brain networks, increased levels of between-network functional connectivity, and an overall more diverse set of neural interactions.

Carhart-Harris et al. (2014) point out a number of implications of EBT. First, they map the feelings of ‘uncertainty’ that often accompany psychedelic effects onto the fact that a more entropic brain dynamic is the information-theoretic equivalent to a more ‘uncertain’ brain dynamic. “Thus, according to the entropic brain hypothesis, just as normally robust principles about the brain lose definition in primary states, so confidence is lost in ‘how the world is’ and ‘who one is’ as a personality” (Carhart-Harris et al., 2014, p. 16).

Second, like Huxley’s cerebral reducing valve and Freud’s ego, EBT argues that the DMN’s organizational stronghold over brain activity can be both an evolutionary advantage *and* a source of pathology. “It is argued that this entropy-suppressing function of the human brain serves to promote realism, foresight, careful reflection and an ability to recognize and overcome wishful and paranoid fantasies. Equally however, it could be seen as exerting a limiting or narrowing influence on consciousness” (Carhart-Harris et al., 2014, p. 7). Carhart-Harris et al. (2014) point out that neuroimaging studies have implicated increased DMN activity and RSFC with various aspects of depressive rumination, trait neuroticism, and depression. “The suggestion is that increased DMN activity and connectivity in mild depression promotes concerted introspection and an especially diligent style of reality-testing. However, what may be gained in mild depression (i.e., accurate reality testing) may be offset by a reciprocal decrease in flexible or divergent thinking (and positive mood)” (Carhart-Harris et al., 2014, p. 10).

Third, consistent with both psychoanalytic and filtration theory, is the notion that psychedelic drugs’ capacity to temporarily weaken, collapse, or disintegrate the normal ego/DMN stronghold underpins their therapeutic utility. “Specifically, it is proposed that psychedelics work by dismantling reinforced patterns of negative thought and behavior by breaking down the stable spatiotemporal patterns of brain activity upon which they rest” (Carhart-Harris et al., 2014, p. 1).

Fourth and finally, EBT sheds light on the shared descriptive elements between psychedelic effects and psychotic symptoms by characterizing both in terms of elevated levels of entropy and uncertainty in brain activity which lead to a “regression” into primary consciousness. The collapse of the organizing effect of DMN coupling and anticorrelation patterns, according to EBT, point to “system-level mechanics of the psychedelic state as an exemplar of a regressive style of cognition that can also be observed in REM sleep and early psychosis” (Carhart-Harris et al., 2014, p. 5).

Thus, EBT formulates all four of the theoretical features identified in filtration and psychoanalytic accounts, but does so using 21st-century empirical data plugged into the quantitative concepts of entropy, uncertainty, criticality, and functional connectivity. EBT hints at possible ways to close the gaps in understanding by offering quantitative concepts that link phenomenology to brain activity and pathogenesis to therapeutic mechanisms.

### Integrated Information Theory

**FIGURE 4:**
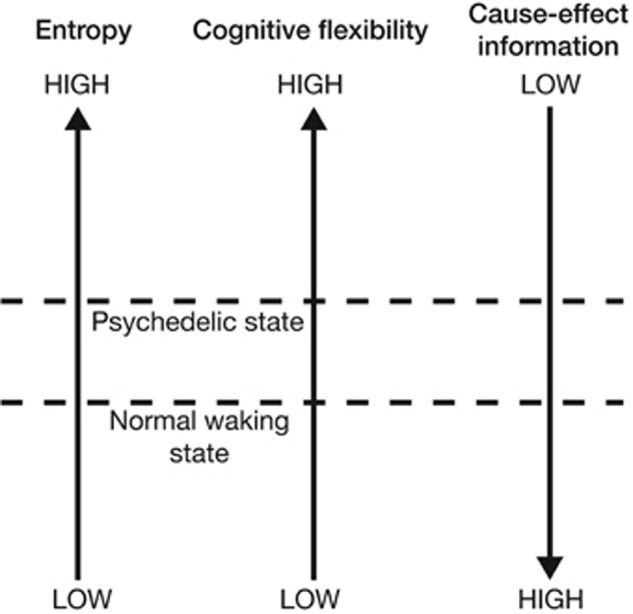
“Increasing neural entropy elevates cognitive flexibility at the expense of a decrease in the cause-effect information specified by individual mechanisms” (Gallimore, 2015, p. 10).

Integrated Information Theory (IIT) is a general theoretical framework which describes the relationship between consciousness and its physical substrates (Oizumi et al., 2014; Tononi, 2004, 2008). While EBT is already loosely consistent with the core principles of IIT, Gallimore (2015) demonstrates how EBT’s hypotheses can be operationalized using the technical concepts of the IIT framework. Using EBT and recent neuroimaging data as a foundation, Gallimore develops an IIT-based model of psychedelic effects. Consistent with EBT, this IIT-based model describes the brain’s continual challenge of minimizing entropy while retaining flexibility. Gallimore formally restates this problem using IIT parameters: brains attempt to optimize the give-and-take dynamic between *cause-effect information* and cognitive flexibility. In IIT, a (neural) system generates cause-effect information when the mechanisms which make up its current state *constrain* the set of states which could casually precede or follow the current state. In other words, each mechanistic state of the brain: (1) limits the set of past states which could have causally given rise to it, and (2) limits the set of future states which can causally follow from it. Thus, each current state of the mechanisms within a neural system (or subsystem) has an associated *cause-effect repertoire* which specifies a certain amount of cause-effect information as a function of how stringently it constrains the unconstrained state repertoire of all possible system states. Increasing the entropy within a cause-effect repertoire will in effect constrain the system less stringently as the causal possibilities are expanded in both temporal directions as the system moves closer to its unconstrained repertoire of all possible states. Moreover, increasing the entropy within a cause-effect repertoire equivalently increases the uncertainty associated with its past (and future) causal interactions. Using this IIT-based framework, Gallimore (2015) argues that, compared with normal waking states, psychedelic brain states exhibit higher entropy, higher cognitive flexibility, but lower cause-effect information (**Figure 4**).

Neuroimaging data suggests that human brains exhibit a larger overall repertoire of neurophysiological states under psychedelic drugs, exploring a greater diversity of states in a more random fashion. For example, in normal waking states, DMN activity ‘rules out’ the activity of TPNs, and vice versa, due to their relatively strict anticorrelation patterns. Brain network anticorrelation generates cause-effect information because it places constraints on the possible causal interactions within and between brain mechanisms; for example, DMN-TPN anticorrelation patterns ‘rule out’ the DMN activity in the presence of activated TPNs. However, psychedelic drugs ‘dissolve’ DMN-TPN (and other) network anticorrelation patterns, which permits simultaneous activation of brain networks which are normally mutually exclusive. The cause-effect repertoire of brain mechanisms thus shifts closer to the unconstrained repertoire of all possible past and future states. This has the effect of “increasing the probability of certain states from zero or, at least, from a very low probability” (Gallimore, 2015, p. 7). Therefore the subjective contents perception and cognition become more diverse, more unusual, and less predictable. This increases flexibility but decreases precision and control as the subjective boundaries which normally demarcate distinct cognitive concepts and perceptual objects dissolve. Gallimore leverages IIT in an attempt unify these phenomena under a formalized framework.

However, as Gallimore notes, “this model does not explain how neural entropy is increased by (psychedelic drugs), but predicts consequences of the entropy increase revealed by functional imaging data” (Gallimore, 2015, p. 7). How do psychedelic drugs increase neural entropy?

### Predictive Processing

The first modern brain imaging measurements in humans under psilocybin yielded somewhat unexpected results: *reductions* in oscillatory power (MEG) and cerebral blood flow (fMRI) correlated with the intensity of subjective psychedelic effects (Carhart-Harris et al., 2012; Muthukumaraswamy et al., 2013). In their discussion, the authors suggest that their findings, although surprising through the lens of commonly held beliefs about how brain activity maps to subjective phenomenology, may actually be consistent with a theory of brain function known as the *free energy principle* (FEP; Friston, 2010).

In one model of global brain function based on the free-energy principle (Friston, 2010), activity in deep-layer projection neurons encodes top-down inferences about the world. Speculatively, if deep-layer pyramidal cells were to become hyperexcitable during the psychedelic state, information processing would be biased in the direction of inference—such that implicit models of the world become spontaneously manifest—intruding into consciousness without prior invitation from sensory data. This could explain many of the subjective effects of psychedelics (Muthukumaraswamy et al., 2013, p. 15181).

What is FEP? “In this view, the brain is an inference machine that actively predicts and explains its sensations. Central to this hypothesis is a probabilistic model that can generate predictions, against which sensory samples are tested to update beliefs about their causes” (Friston, 2010). FEP is a formulation of a broader conceptual framework emerging in cognitive neuroscience known as *predictive processing* (PP; Clark, 2013)^10^. PP has links to *bayesian brain hypothesis* (Knill and Pouget, 2004), *predictive coding* (Rao and Ballard, 1999), and earlier theories of perception and cognition (MacKay, 1956; Neisser, 1967; Gregory, 1968) dating back to Helmholtz (1925) who was inspired by Kant (1996; see Swanson, 2016). At the turn of the 21st century, the ideas of Helmholtz catalyzed innovations in machine learning (Dayan et al., 1995), new understandings of cortical organization (Mumford, 1992; Friston, 2005), and theories of how perception works (Kersten and Yuille, 2003; Lee and Mumford, 2003).

PP subsumes key elements from these efforts (see Clark, 2013) to describe a universal principle of brain function captured by the idea of *prediction error minimization* (PEM; Hohwy, 2013). What does it mean to say that the brain works to minimize its own prediction error? Higher-level areas of the nervous system (i.e., higher-order cortical structures) generate top-down synaptic ‘predictions’ aimed at matching the expected bottom-up synaptic activity at lower-level areas, all the way down to ‘input’ activity at sense organs. Top-down signals encode a kind of ‘best guess’ about the most likely (hidden)^11^ causes of bodily sensations. In this multi-level hierarchical cascade of neural activity, high-level areas attempt to ‘explain’ the states of levels below via synaptic attempts to *inhibit* lower-level activity—“high-level areas tell lower levels to ‘shut up”’ (Kersten et al., 2004, p. 297). But lower levels will not ‘shut up’ until they receive top-down feedback (inference) signals that adequately fit (explain) the bottom-up (evidence) signals. Mismatches between synaptic ‘expectation’ and synaptic ‘evidence’ generate *prediction error signals* which ‘carry the news’ by propagating the ‘surprise’ upward to be ‘explained away’ by yet higher levels of hierarchical cortical processing anatomy (see Clark, 2015). This recurrent neural processing scheme approximates (empirical) Bayesian inference (Friston and Stephan, 2007) as the brain continually maps measured bodily effects to different sets of possible causes and attempts to select the set of possible causes that can best ‘explain away’ the measured bodily effects. Crucially, the sets of possible causes must be *narrowed* in order for the system to settle on an explanation (Tenenbaum et al., 2011). Prior constraints which allow the system to narrow the hypothesis space are known as ‘inductive biases’ or *priors* (Kemp et al., 2007; Tenenbaum et al., 2011; Clark, 2013). Efforts in Bayesian statistics and machine learning have demonstrated that improvements in inductive capabilities occur when priors are linked in a multi-level hierarchy, with “not just a single level of hypotheses to explain the data but multiple levels: hypothesis spaces of hypothesis spaces, with priors on priors” (Tenenbaum et al., 2011, p. 1282). Certain priors in the hierarchy, known as ‘hyperpriors’ (Friston et al., 2013) or ‘overhypotheses’ (Goodman, 1983; Kemp et al., 2007) are more abstract and allow the system to ‘rule out’ large swaths of possibilities, drastically narrowing the hypothesis space, making explanation more tractable (Blokpoel et al., 2012). For example, the brute constraints of space and time act as hyperpriors; e.g., prior knowledge “that there is only one object (one cause of sensory input) in one place, at a given scale, at a given moment,” or the fact that “we can only perform one action at a time, choosing the left turn or the right but never both at once” (Clark, 2013, p. 196).

Thus, PP states that brains are neural generative models built from linked hierarchies of priors where higher levels continuously attempt to ‘guess’ and explain activity at lower levels. The entire process can be characterized as the agent’s attempt to *optimize* its own internal model of the sensorium (and the world) over multiple spatial and temporal scales (Friston, 2010).

Interestingly, PP holds that our perceptions of external objects recruit the same synaptic pathways that enable our capacity for *mental imagery, dreaming, and hallucination*. The brain’s ability to ‘simulate’ its own ‘virtual reality’ using internal (generative) models of the world’s causal structure is thus crucial to its ability to perceive the external world. “[A] fruitful way of looking at the human brain, therefore, is as a system which, even in ordinary waking states, constantly hallucinates at the world, as a system that constantly lets its internal autonomous simulational dynamics collide with the ongoing flow of sensory input, vigorously dreaming at the world and thereby generating the content of phenomenal experience” (Metzinger, 2003).

How do psychedelic molecules perturb predictive processing? If normal perception is a kind of ‘controlled hallucination’ (see Clark, 2015) where top-down simulation is constrained by bottom-up sensory input colliding with priors upon priors, then, as the above quotation from Muthukumaraswamy et al. (2013) suggests, psychedelic drugs essentially cause perception to be *less controlled* hallucination. The idea is that psychedelic drugs perturb the (learned and innate) prior constraints on internal generative models. Via their 5-HT_2A_ agonism, psychedelic drugs cause hyperexcitation in layer V pyramidal neurons, which might cause endogenous simulations to ‘run wild’ so that awareness becomes more imaginative, dreamlike, and hallucinatory. This hypothesis could in principle still be consistent with observed *reductions* in brain activity under psychedelics; recall from above that, in PP schemes, the higher-level areas ‘explain away’ lower-level excitation by *suppressing it with top-down inhibitory signals*. “Here, explaining away just means countering excitatory bottom-up inputs to a prediction error neuron with inhibitory synaptic inputs that are driven by top-down predictions” (Friston, 2010, p. 130).

How does PP tie into filtration theories and psychoanalytic accounts? Carhart-Harris et al. (2012) link Huxley with Friston to interpret their initially surprising fMRI scans of humans under psilocybin (see also Zizo, 2013). One objection to this linkage might be that Huxley often describes psychedelic opening of the cerebral reducing valve as revealing more of the *world*. At first glance this seems at odds with the above PP account of psychedelic effects, which describes psychedelic drugs causing rampant *internal* simulations of reality, not revealing more of the external world. However, this apparent tension might be resolved in light of *active inference*, a key principle of FEP (Friston, 2010). Active inference shows how internal models do not merely generate top-down (inference) signals but also shape the *sampling* and *accumulation* of bottom-up sensory (evidence) signals. “In short, the agent will selectively sample the sensory inputs that it expects. This is known as active inference. An intuitive example of this process (when it is raised into consciousness) would be feeling our way in darkness: we anticipate what we might touch next and then try to confirm those expectations” (Friston, 2010, p. 129). The principle of active inference hints at a resolution to the apparent tensions between Osmond’s ‘mind-manifesting’ model and Huxley’s ‘world-manifesting’ model. Psychedelics manifest *mind* by perturbing prior constraints on internal generative models, thereby expanding the possibilities in our inner world of feelings, thoughts, and mental imagery. Importantly, this could also manifest normally ignored aspects of *world* by altering active inference, which would in effect expand the sampling of sensory data to include samples that are normally routinely ‘explained away.’ Potentially, this understanding goes some way in explaining the perception-hallucination continuum of psychedelic drug effects (reviewed above) as it shows how perceptual *intensifications*, on the one hand, and *distortions and hallucinations*, on the other hand, could both be caused by a synaptic disruption of hierarchically linked priors in internal generative models.

The brief speculative remark by Muthukumaraswamy et al. (2013) is not the only PP-based account of psychedelic drug effects. The PP framework describes a recurrent back-and-forth give-and-take between colliding top-down and bottom-up signals, where internal models serve to shape experience and experience serves to build internal models, so this leaves room for rival PP-based accounts that diverge regarding where exactly the psychedelic drug perturbs the system. For example, increased top-down activity could be the result of pharmacological hyperactivation of top-down synaptic transmission; yet equally plausible is the hypothesis that increased top-down activity is a *compensatory response* to pharmacological attenuations or distortions of bottom-up signal.

For example, Corlett et al. (2009, p. 521) hypothesize that LSD hallucinations result from “noisy, unpredictable bottom-up signaling in the context of preserved and perhaps enhanced top-down processing.” In contrast to the PP-based account outlined above, which focuses on changes to top-down signals, the strategy of Corlett et al. (2009) is to map various psychedelic effects to disturbances of top-down *and/or* bottom-up signals. The issue of what is primary and what is compensatory illustrates the vast possibilities in the hypothesis space of PP-based accounts.

While most PP-based accounts point to changes in top-down signaling, even within this hypothesis space there are contrasting conceptions of exactly how psychedelic molecules perturb top-down processing. Briefly, these differing hypotheses include: (1) *hyperactivation* or *heavier weighting* of top-down signaling (Muthukumaraswamy et al., 2013; described above), (2) *reduced* influence of signals from higher cortical areas (Carhart-Harris and Friston, 2010; McKenna and Riba, 2015), (3) interference with *multisensory integration* processes and PP-based binding of sensory signals (Carhart-Harris and Friston, 2010; Letheby and Gerrans, 2017; Millière, 2017), and (4) changes in the *composition* and *level of detail* specified by top-down signals (Pink-Hashkes et al., 2017).

Carhart-Harris and Friston (2010) argue that the Freudian conception of ego, with its organizing influence over the primary process, is consistent with PP descriptions of higher-level cortical structures predicting and suppressing the excitation in lower levels in the hierarchy (i.e., limbic regions). Freud hypothesized that the secondary process binds, integrates, and organizes the ‘lower’ and more chaotic neural activity of the primary process into the broader and more cohesive composite structure of the ego. Carhart-Harris and Friston (2010) argue that when large-scale intrinsic networks become dis-integrated, the activity at lower levels can no longer be ‘explained away’ (suppressed) by certain higher-level systems, causing conscious awareness to take on hallmark characteristics of the primary process. In normal adult waking states, networks based in higher-level areas can successfully predict and explain (suppress and control) the activity of lower level areas. “In non-ordinary states, this function may be perturbed (e.g., in the case of hallucinogenic drugs, through actions at modulatory post-synaptic receptors), compromising the hierarchical organization and suppressive capacity of the intrinsic networks” (Carhart-Harris and Friston, 2010, p. 1274).

Similar PP-based theories of psychedelic ego dissolution have been proposed without invoking Freud (Letheby and Gerrans, 2017; Millière, 2017). PP posits that the brain explains self-generated stimuli by attributing its causes to a coherent and persisting entity (i.e., the self), much like how it predicts and explains external stimuli by attributing their causes to coherent and persisting external objects (see also Limanowski and Blankenburg, 2013; Allen and Friston, 2016; Letheby and Gerrans, 2017; Millière, 2017). Letheby and Gerrans (2017) use the PP framework to recast the psychoanalysis-based theories of LSD ego effects proposed by Savage (1955)^12^ and Klee (1963) described in Section “Psychoanalytic Theory.” The core idea is that psychedelic drugs interfere with processes that bind and integrate stimuli according to probabilistic estimates of how relevant the stimuli are to the organism’s (self) goals. Letheby and Gerrans (2017, p. 7) point out that ego dissolution under psychedelic drugs is correlated with the desynchronization (reductions in intrinsic functional connectivity) of brain networks implicated in “one aspect or another of self-representation”—specifically the salience network (SLN) and the DMN (Tagliazucchi et al., 2016). This causes an ‘unbinding’ of stimuli that are normally processed according to self-binding multisensory integration mechanisms. “Attention is no longer guided exclusively by adaptive and egocentric goals and agendas; salience attribution is no longer bound to personal concern” (Letheby and Gerrans, 2017, p. 6). This description echoes Huxley’s cerebral reducing valve “in which the brain with its associated *normal self*, acts as a utilitarian device for limiting, and making selections from, the enormous possible world of consciousness, and for canalizing experience into biologically profitable channels” (Huxley, 1999, p. 29; emphasis mine). Letheby and Gerrans’ PP-based account elucidates how psychedelic drugs could perturb the brain’s “associated normal self” preventing the usual self-binding of internal and external stimuli.

Pink-Hashkes et al. (2017, p. 2907) argue that under psychedelic drugs “top-down predictions in affected brain areas break up and decompose into many more overly detailed predictions due to hyper activation of 5-HT_2A_ receptors in layer V pyramidal neurons.” Pink-Hashkes et al. (2017) state that when internal generative models are described as categorical probability distributions rather than Gaussian densities (Friston et al., 2015; Kwisthout et al., 2017), “the *state space granularity* (how detailed are the generative models and the predictions that follow from them) is crucial” (Kwisthout et al., 2017, p. 2; see also Kwisthout and van Rooij, 2015). Categorical predictions that are less detailed will ‘explain’ more bottom-up data (because they cover more ground) and thus produce less prediction error. Categorical predictions that are more detailed, by contrast, will carry less precision and thus potentially generate more prediction error (Kwisthout and van Rooij, 2015; Kwisthout et al., 2017). Pink-Hashkes et al. (2017, p. 2908) propose that psychedelic drugs cause brain structures at certain levels of the cortical hierarchy to issue more detailed (less abstract) ‘decomposed’ predictions that “fit less data than the ‘usual’ broad prediction.” They argue that many psychedelic effects stem from the brain’s attempts to *compensate* for these decomposed top-down predictions as it responds to the increase in prediction errors that result from overly detailed predictions.

In summary, the current state of PP-based theories of psychedelic effects reveals a divergent mix of heterogeneous ideas and conflicting hypotheses. Do psychedelic molecules perturb top-down (feedback) signaling, or bottom-up (feedforward) signaling, or both? Do the subjective phenomenological effects result from direct neuropharmacological changes or compensatory mechanisms responding to pharmacological perturbations? Yet there seems to be a core intuition that transcends the conceptual variance here: psychedelic drugs (somehow) interfere with established priors that normally constrain the brain’s internal generative models.

Predictive processing-based accounts, consistent with EBT and IIT (and filtration and psychoanalytic accounts), propose that psychedelic drugs disrupt neural mechanisms (priors on internal generative models) which normally constrain perception and cognition. Perturbing priors causes subjective phenomenology to present a wider range of experiences with increased risk of perceptual instability and excessive cognitive flexibility. As prior constraints on self and world are loosened, the likelihood of psychosis-like phenomena increases. At the same time, novel thinking is increased and the brain becomes more malleable and conducive to therapeutic cognitive and behavioral change.

## Conclusion

The four key features identified in filtration and psychoanalytic accounts from the late 19th and early 20th century continue to operate in 21st-century cognitive neuroscience: (1) psychedelic drugs produce their characteristic diversity of effects because they perturb adaptive mechanisms which normally constrain perception, emotion, cognition, and self-reference, (2) these adaptive mechanisms can develop pathologies rooted in either too much or too little constraint (3) psychedelic effects appear to share elements with psychotic symptoms because both involve weakened constraints (4) psychedelic drugs are therapeutically useful precisely because they offer a way to temporarily inhibit these adaptive constraints. It is on these four points that EBT, IIT, and PP seem consistent with each other and with earlier filtration and psychoanalytic accounts. EBT and IIT describe psychedelic brain dynamics and link them to phenomenological dynamics, while PP describes informational principles and plausible neural information exchanges which might underlie the larger-scale dynamics described by EBT and IIT. Certain descriptions of neural entropy-suppression mechanisms (EBT), cause-effect information constraints (IIT), or prediction-error minimization strategies (PP, FEP) are loosely consistent with Freud’s ego and Huxley’s cerebral reducing valve.

In surveying the literature for this review I can confidently conclude that 21st-century psychedelic science has yet to approach a unifying theory linking the diverse range of phenomenological effects with pharmacology and neurophysiology while tying these to clinical efficacy. However, the historically necessary ingredients for successful theory unification—formalized frameworks and unifying principles (Morrison, 2000)—seem to be taking shape. Formal models are an integral part of 21st-century neuroscience (Forstmann et al., 2011) where they help to describe natural principles in the brain and aid explanation and understanding (Kay, 2017).^13^ Here I have reviewed a handful of formalized frameworks—EBT, IIT, PP—which are just beginning to be used to account for psychedelic effects. I have also highlighted the fact that all of the accounts reviewed here, from the 19th-century to the 21st-century, propose that psychedelic drugs inhibit neurophysiological constraints in order to produce their diverse phenomenological, psychotomimetic, and therapeutic effects.

Why should we pursue a unified theory of psychedelic drug effects at all? To date, theories of brain function and mind in general have resisted the kind of unification that has occurred in other areas of science (Huang, 2008; Edelman, 2012). Because the human brain has evolved disparate and complex layers under diverse environmental circumstances, many doubt the possibility of and debate the merits of seeking ‘grand unified theories’ (GUTs) of brain function. “There is every reason to think that there can be no grand unified theory of brain function because there is every reason to think that an organ as complex as the brain functions according to diverse principles” (Anderson and Chemero, 2013, p. 205). Indeed, Anderson and Chemero (2013, p. 205) caution that “we should be skeptical of any GUT of brain function” and charge that PP in particular, when taken as a unified theory as outlined by Clark (2013), “threatens metaphysical disaster.”

Given these understandable critical reservations about seeking after GUTs of brain function (and therefore any truly unifying theory of psychedelic drug effects), it is perhaps safer to aspire for theories that feature “broad explanatory frameworks” and offer “conceptual breadth” allowing us to “paint the big picture” (Edelman, 2012). PP and FEP, at the very least, offer a broad explanatory framework that emcompasses a large swath of perceptual and cognitive phenomena (Huang, 2008; Friston, 2010; Clark, 2015). Psychedelic drugs offer a unique way to iteratively develop and test such big-picture explanatory frameworks: these molecules can be used to probe the links between neurochemistry and neural computation across multiple layers of neuroanatomy and phenomenology. Meeting the challenge of predicting and explaining psychedelic drug effects is the ultimate acid test for any unified theory of brain function.

## Author Contributions

LS researched and wrote the manuscript.

## Conflict of Interest Statement

The author declares that the research was conducted in the absence of any commercial or financial relationships that could be construed as a potential conflict of interest.

## Footnotes

1 For example, see Kitcher (1981, 1989), Friedman (1983), and Morrison (2000).

2 Ayahuasca contains DMT but is importantly different from pure DMT (McKenna et al., 1984).

3 Variously termed ‘ego disintegration,’ ‘ego loss,’ and ‘ego death.’ For a comprehensive review, see Millière (2017).

4 An unnamed JAMA book reviewer critically notes that “it is interesting that attention had not been paid by American scientists to this intoxicant used by the Mexican Indians until a European called attention to it” (Beringer, 1927a).

5 A marketing team at Sandoz Pharmaceuticals sent free samples of LSD to physicians around the world and inside each package was a pamphlet which read: “By taking Delysid [LSD] himself, the psychiatrist is able to gain an insight into the world of ideas and sensations of mental patients. Delysid can also be used to induce model psychoses of short duration in normal subjects, thus facilitating studies on the pathogenesis of mental disease” (Hofmann, 1980, p. 47).

6 In this sense LSD catalyzed the neuroscientific revolution of serotonin neurochemistry (Nichols, 2016) and crystallized the emergence of the field of neuropharmacology.

7 In fact, in the terminology of biological science, a *model* is “an organism whose appearance a mimic imitates” (Merriam-Webster, 2017).

8 Huxley was overtly critical of Freud, yet Huxley’s cerebral reducing valve is strikingly similar to Freud’s ego (see Benton, 2016 for a comparison of Freud and Bergson).

9 “The secondary process is characterized by a bound state in the neurone, which though there is a high cathexis, permits only a small current… Now the ego itself is a mass like this of neurones which hold fast to their activity—are, that is in a bound state and this surely can only happen as a result of the effect they have on one another. We can therefore imagine that a perceptual neurone which is active with attention is as a result temporarily, as it were, taken up into the ego and is now subject to the same binding of its energy as are all the other ego neurones… This bound state, which combines high activity with small current, would thus characterize processes of thought mechanically” (Freud, 1895, p. 368).

10 See also Clark (2015) and Wiese and Metzinger (2017) for introductory reviews conceptual overviews.

11 The causes of our bodily sensations cannot be directly observed by the brain: an organism’s brain is ‘skull-bound’ (Hohwy, 2013) and limited to a ‘view from inside the black box’ (Clark, 2013).

12 “Disturbances in perception caused by LSD make it impossible for the ego to integrate the evidence of the senses and to coordinate its activities …” (Savage, 1955, p. 14).

13 This remains true regardless of the outcome of healthy debates about the nature and proper use of models in science (Frigg and Hartmann, 2017).
